# Layering vaccination with antibiotic therapy results in protection and clearance of *Burkholderia pseudomallei* in Balb/c mice

**DOI:** 10.1128/iai.00455-23

**Published:** 2024-01-30

**Authors:** Kay B. Barnes, Paul Brett, Mary Burtnick, Andreas Vente, Christine Bentley, Mark I. Richards, Helen C. Flick-Smith, Gary Burgess, Joanne E. Thwaite, Thomas R. Laws, Thomas C. Maishman, Michelle Nelson, Sarah V. Harding

**Affiliations:** 1 Defence Science and Technology Laboratory, Porton Down, Salisbury, United Kingdom; 2 University of Nevada, Reno School of Medicine, Reno, Nevada, USA; 3 Mahidol University, Bangkok, Thailand; 4 MerLion Pharmaceuticals, Berlin, Germany; 5 University of Leicester, Leicester, United Kingdom; Washington State University, Pullman, Washington, USA

**Keywords:** melioidosis, finafloxacin, layered defence, subunit vaccine, Balb/c, *Burkholderia pseudomallei*

## Abstract

Melioidosis is a disease that is difficult to treat due to the causative organism, *Burkholderia pseudomallei* being inherently antibiotic resistant and it having the ability to invade, survive, and replicate in an intracellular environment. Combination therapy approaches are routinely being evaluated in animal models with the aim of improving the level of protection and clearance of colonizing bacteria detected. In this study, a subunit vaccine layered with the antibiotic finafloxacin was evaluated *in vivo* against an inhalational infection with *B. pseudomallei* in Balb/c mice. Groups of mice vaccinated, infected, and euthanized at antibiotic initiation had a reduced bacterial load compared to those that had not been immunized. In addition, the subunit vaccine provided a synergistic effect when it was delivered with a CpG ODN and finafloxacin was initiated at 48 h post-challenge. Vaccination was also shown to improve the outcome, in a composite measure of survival and clearance. In summary, layering a subunit vaccine with the antibiotic finafloxacin is a promising therapeutic alternative for use in the treatment of *B. pseudomallei* infections.

## INTRODUCTION

*Burkholderia pseudomallei* is a Gram negative bacterium which is the causative agent of melioidosis, a difficult disease to treat due to it being inherently resistant to antibiotics and the ability of the bacterium to invade and survive within cells, avoiding some aspects of the immune system (1). Melioidosis is known to be endemic in many parts of the world including South East Asia and Northern Australia; however, it is possible that *B. pseudomallei* is endemic in many other regions where clinical and laboratory resources are limited and where it has not yet been identified (2). Recent reports have estimated that the number of cases of melioidosis per year is as high as 165,000, with 89,000 deaths (3).

The clinical presentation of melioidosis is wide ranging and is dependent on many factors including the route of infection, virulence, and infectious dose of the bacterial strain and host factors including conditions for example diabetes, alcoholism, and renal conditions (4). Pneumonia often associated with bacteremia is common, but other clinical manifestations can include abscess formation, skin and soft tissues, musculoskeletal and neurological involvement, typically dependent on the route of exposure (1, 5).

There is currently no licensed vaccine for melioidosis although many studies have investigated the use of killed bacterial cells, live attenuated strains, and subunit approaches (6–10). One of the leading candidates that has been extensively evaluated in mouse models and will soon be moving into Phase 1 trials is a capsular polysaccharide (CPS)-based subunit vaccine. The CPS component is isolated from the non-pathogenic surrogate organism, *Burkholderia thailandensis*, and conjugated to the carrier protein CRM197, a non-toxic mutant of diphtheria toxin, used in many polysaccharide vaccines including those against *Neisseria meningitidis* and *Streptococcus pneumoniae* to boost the immune response generated to the polysaccharide antigen (11). In addition, the recombinant protein, haemolysin co-regulated protein 1 (Hcp1), a component of the Type VI secretion system, is included in the vaccine. Hcp1 has been shown to improve the immune response and is correlated with protection in human cases of melioidosis (12, 13).

CPS-CRM197 combined with Hcp1 has demonstrated complete protection in a C57BL/6 mouse model, which is thought to be more “human like” in terms of Th1/Th2 immune bias (14). In addition, this subunit vaccine conferred a high level of clearance (70% of surviving animals were clear of colonizing bacteria). The Balb/c mouse is considered to be a good model for the evaluation of therapeutics, but not vaccines, that rely on a Th1 biased response, a CPS glycoconjugate vaccine delivered with the recombinant ABC transporter LolC, offering protection when infected by the intraperitoneal route (15, 16). It was hypothesized that the addition of a suboptimal antibiotic regimen (achieved by delivering fewer doses) in a layered defense approach may provide synergy, resulting in high levels of protection while improving clearance of colonizing bacteria from tissues.

Currently, the treatment of melioidosis is 14 days of intravenously administered ceftazidime or meropenem followed by an oral eradication phase of at least 12 weeks, usually co-trimoxazole or co-amoxiclav (17). The most recent review of the 2015 Darwin melioidosis guidelines still recommend this regimen with the addition of increasing the intravenous antibiotic phase for 3 or 4 weeks for patients with bacteraemia and pneumonia, in particular multi-lobar pneumonia (17). With the emergence of naturally evolving resistance to commonly used antibiotics and the potential of relapse and recrudescence of infection in people, the investigation of different therapeutic interventions is warranted.

One such therapeutic is the novel C-8-cyano-fluoroquinolone finafloxacin, which has demonstrated enhanced activity under acidic conditions, often found at the site of bacterial infection and where other fluoroquinolones, including ciprofloxacin, are less active (18, 19). Finafloxacin is being developed by MerLion Pharmaceuticals GmbH for the treatment of serious infections with acidic foci and has obtained approval by the US FDA and Health Canada as a topical medication for the treatment of acute otitis (18, 20, 21). Finafloxacin has been extensively evaluated in preclinical and clinical studies which have shown that finafloxacin has increased bactericidal activity at infection-relevant acidic pH against multiple Gram negative and Gram positive bacterial species including *Legionella pneumophila*, ciprofloxacin-resistant strains of *Escherichia coli* and *Acinetobacter baumannii,* and large strain panels of respiratory pathogens (18, 19, 22–26). Furthermore, we have demonstrated that finafloxacin has *in vitro* activity and *in vivo* efficacy against a range of biothreat pathogens including *Yersinia pestis, Francisella tularensis, Bacillus anthracis, Burkholderia mallei, Coxiella burnetii,* and *B. pseudomallei* (27–32).

Although a number of antibiotics including finafloxacin have been shown to offer some protection in mouse models of melioidosis, a number of animals still succumb to disease following a relapse of infection. A number of different combination therapy approaches have been evaluated to treat *B. pseudomallei* infections in mice, with the aim of identifying candidates, that when layered, offer a high level of protection, clearance of colonizing bacteria from tissues, and a reduction in the rate of relapse of infection observed. Previous studies have detailed the benefits of combining antibiotics, antibiotics with immunomodulators, and vaccines with antibiotics (33–35). In addition, the CPS-based subunit vaccine evaluated in this manuscript has been utilized as a component of a combination approach in a similar study with co-trimoxazole in the C57BL/6 mouse model of melioidosis. This study evaluated the subunit vaccine in combination with 7 or 21 days of co-trimoxazole treatment (35). This study resulted in 90%–100% protection when 7 or 21 days of co-trimoxazole were delivered (dependent on the time of antibiotic initiation) (35).

The aim of the study detailed in this manuscript was to evaluate layering the CPS-CRM197 delivered with Hcp1, shown to be suboptimal in the Balb/c mouse model, with a short course of antibiotics (7 days of finafloxacin compared to 14 days) to investigate whether this combination therapy approach had a positive benefit in terms of protection offered, clearance of colonizing bacteria from tissues and the incidence of relapse of disease. In addition, the inclusion of a CpG ODN as a component of the adjuvant was compared to the vaccine delivered without this component.

## MATERIALS AND METHODS

### Bacteria

*B. pseudomallei* strain K96243 was streaked onto Luria agar (L-agar) and incubated at 37°C for 24 h. The following day, a loopful of bacteria was re-suspended into 10 mL of phosphate-buffered saline (PBS) and adjusted to an OD_590nm_ of 0.36. 1 mL of this was inoculated into 100 mL of L-broth. This was incubated at 37°C with shaking at 180 rpm for 16 h and adjusted to an OD_590nm_ of 0.36 in 10 mL of PBS to obtain approximately 1 × 10^8^ CFU/mL. A 1:50 dilution was performed in PBS, which was used in the efficacy study.

### Vaccine

All components of the vaccine used in the efficacy study were provided by the University of Nevada, Reno (UNR). The 6-deoxyheptan capsular polysaccharide (CPS) was purified from *Burkholderia thailandensis* strain BT2683 (an OPS-deficient derivative of strain E555), chemically activated, and covalently linked to the recombinant CRM197 diphtheria toxin mutant (CRM197) to produce CPS-CRM197 (14). Recombinant *Burkholderia mallei* Hcp1 was also added to the formulation (36).

### Adjuvants

CpG (ODN 2006) was purchased from Invivogen (Toulouse, France) and reconstituted in 1 mL of sterile endotoxin free water to produce a 1 mg/mL solution. Alhydrogel 2% was also purchased from Invivogen (Toulouse, France).

### Antibiotic

Finafloxacin in salt form was supplied by MerLion Pharmaceuticals Ltd. A 15 mg/mL solution of finafloxacin was prepared by adding 2.1 mL of 0.01M Tris buffer to 44 mg of finafloxacin powder (containing 37.5 mg of active ingredient). Two-hundred microliters of 1 M sodium hydroxide was added to dissolve the antibiotic followed by 200 µL of 0.01 M hydrochloric acid. The pH of the resulting solution was pH 8.

### Mice

Animal studies were carried out in accordance with the UK Animals (Scientific Procedures) Act 1986 and an ACURO appendix. Female BALB/c mice (Charles River Laboratories, UK) aged 6–8 weeks were randomized into cages of 5, stored within a rack during the vaccination procedures, and then moved to a Class III half suit rigid-walled isolator in an ACDP Containment Level 3 laboratory for the efficacy study. Mice had free access to water and rodent diet (Harlan Teklad, UK) and underwent an acclimatization period of 6 days before any procedures were performed.

### Vaccinations

Mice were immunized three times by the subcutaneous route (on days 0, 21, and 35) with the CPS-CRM197, Hcp1, Alhydrogel, and CpG (vaccine with CpG); CPS-CRM197, Hcp1, and Alhydrogel (vaccine); adjuvant only (Alhydrogel with CpG); or PBS. Each mouse received 0.25 µg of CPS (as a conjugate), 0.5 µg of Hcp 1, 250 µg of Alhydrogel, and 10 µg of CpG (if required) per dose. Blood samples were collected via a superficial tail vein 1 week prior to challenge, centrifuged at 3,000 rpm for 15 min, and the sera removed and used in the ELISAs to determine the antibody titers against the CPS and Hcp1.

### ELISAs

U96 Maxisorp Nunc-Immuno plates were coated with 100 µL/well of 1 µg/mL (100 ng/well) of CPS or Hcp1 in carbonate coating buffer and were incubated overnight at 4°C. Plates were washed with Tris-buffered saline and tween (TBS-T) (20 mL 1 M Tris pH 7.4, 9 g NaCl, 500 µL Tween 20, dH_2_O up to 1 L) three times in a plate washer. Plates were then blocked with 200 µL/well of room temperature Starting Block T20 Blocking Buffer (SB) and incubated for 30 min. Plates were washed again with TBS-T three times in a plate washer. One-hundred microliters per well of sera harvested from mice by tail bleed (diluted in 10% SB in TBS-T) was added and incubated for 1 h at 37°C. Plates were washed with TBS-T three times in a plate washer, and 100 µL/well of the secondary antibody (goat anti-mouse IgG HRP, Cayman Chemical) diluted 1/4,000 in 10% SB in TBS-T was added and incubated for 1 h at 37°C. Plates were washed with TBS-T, four times in a plate washer. One-hundred microliters per well of tetramethylbenzidine (TMB) Peroxidase Substrate was added, incubated in the dark at room temperature for 20 min, and read at 620 nm on a plate reader.

### Efficacy study

*B. pseudomallei* strain K96243 was aerosolized using a 3-jet Collison nebulizer containing 15 mL of bacteria (prepared as above) controlled and conditioned to an average of 72% ± 2% relative humidity, by an AeroMP platform system (Biaera Technologies, Hagerstown, MD, USA) (37). Animals were exposed (nose only) to the aerosol for a total of 10  min, with sampling achieved for 1 min at the mid-point of the challenge (4.5–5.5 min) using an all-glass impinger (AGI-30; Ace Glass, Vineland, NJ, USA) containing 10 mL PBS. The calculated, presented challenge dose was determined using the bacterial enumerations from the aerosol samples and Guyton’s formula for the respiratory volumes of laboratory animals (20 mL min^−1^) (38). A retained dose of 40% of the presented dose was also calculated (39).

Treatment was initiated at 36 or 48 h post-challenge and continued for 7 days. Finafloxacin (23.1 mg/kg) or the diluent was administered by the oral route three times a day. The dosing regimen used was determined previously through pharmacokinetic profiling to determine delivery of a human equivalent dose (33). Mice were weighed daily and observed a minimum of twice daily for the development of clinical signs of disease for 35 days when the experiment was terminated.

A number of scheduled culls were performed at different time-points throughout the study. The first cull was at treatment initiation (36 or 48 h post-challenge), the second following 7 days of treatment, and finally at the end of the study (day 35 post-challenge). Organs and urine (if the animals urinated at cull point) were harvested and processed for bacterial burden. Blood was also collected from all surviving animals. The study design for the efficacy study is detailed in Table 1.

**TABLE 1:** Study plan for the efficacy study^*a*^

Antibiotic treatment and initiation time	Vaccine	Number for survival	Number for cull	Total number of mice
Finafloxacin (36 h)	None	10	5 at EOT	15
Finafloxacin (36 h)	CPS-CRM197 + Hcp1/Al + CpG	10	5 at EOT	15
Finafloxacin (36 h)	CPS-CRM197 + Hcp1/Al	10	5 at EOT	15
Finafloxacin (48 h)	None	10	5 at EOT	15
Finafloxacin (48 h)	CPS-CRM197 + Hcp1/Al + CpG	10	5 at EOT	15
Finafloxacin (48 h)	CPS-CRM197 + Hcp1/Al	10	5 at EOT	15
Diluent	None	10		10
Diluent	Adjuvant (Al + CpG)	10	5 at 36 h 5 at 48 h	20
Diluent	CPS-CRM197 + Hcp1/Al + CpG	10	5 at 36 h 5 at 48 h 5 at EOT	25
Diluent	CPS-CRM197 + Hcp1/Al	10	5 at 36 h 5 at 48 h 5 at EOT	25
None	None		5 at 36 h 5 at 48 h	10

^*a*^ Al, Alhydrogel; EOT, end of treatment.

### Minimum inhibitory concentration

Bacterial isolates (*n* = 12) were harvested from animals that were treated with finafloxacin and survived until the end of the efficacy study. MICs were carried out on these isolates using E tests (AB Biodisk, Sweden) as per the manufacturer’s instructions.

### Statistical analysis

SPSS (V27.1) was used for these analyses. Graphs were prepared using Graphpad PRISM V8.0. The survival data were initially analyzed using Cox regression. All mice were included in the survival analyses, including those that were euthanized at set time points. The survival of these mice was considered censored beyond the scheduled cull. A main effects model without interactions was fit to the data as imbalances in groups did not allow for interactions. Subsequently, logrank tests were used for individual comparisons, and the Bonferroni’s method was used to account for familywise error for critical comparisons. Antibody titers were compared by the Mann-Whitney test due to the data not being Gaussian. Collett’s model building was used to analyze the bacterial data from animals infected and culled at treatment initiation using a mixed model where model entry was predicated with a probability of P<0.05 of increasing model fit (40). Variables were removed where P>0.15 of reducing the model fit. The mixed model accounted for multiple organs from each individual mouse and the null model included organ (as the bacterial load is known to be dependent on organ). For outcome analysis, only mice observed for the whole experiment were included and outcome was on a three-point scale (2 = euthanized due to lethal endpoint, 1 = survived to the end of the experiment but colonized, or 0 = survived with no detectable bacteria in organs at the end of the experiment). Collett’s model building was also used to analyze the outcome scale data using a generalized ordinal (logit) model where model entry was predicated with a probability of P<0.05 of increasing model fit (40). Variables were removed where P>0.15 of reducing the model fit.

## RESULTS

### Inclusion of CpG as a component of the adjuvant enhances the antibody response to the vaccine

**Fig 1:**
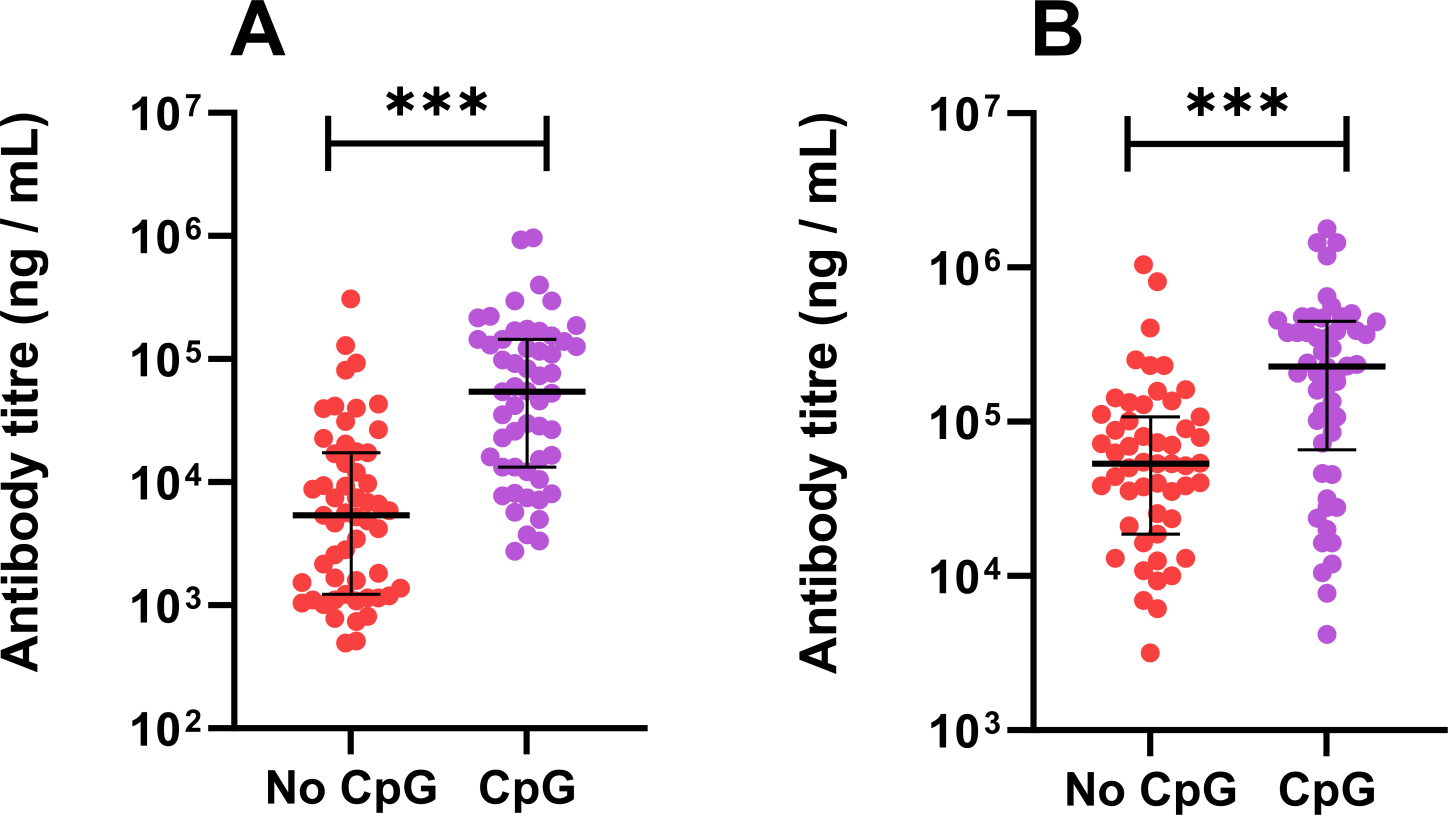
The total IgG titers in mice vaccinated with CPS-CRM197, Hcp1, and Alhydrogel (with and without the CpG). Antibody titers against Hcp1 (**A**) and CPS (**B**) were determined by ELISAs. The Mann-Whitney U test was used to compare the groups (***P<0.001).

Groups of mice were immunized three times with the vaccine formulations, and a week before the bacterial challenge, blood was harvested and sera was analyzed by ELISA for the presence of antibodies to the CPS and Hcp1. No antibody response to either component was observed in any of the unvaccinated mice (n=20 tested). High titers of antibodies against the CPS and Hcp1 were observed in vaccinated mice. Titers were compared between mice immunized with the vaccine (n=54) and mice immunized with the vaccine with CpG, (n=55). The CpG component enhanced the response to both antigens (Fig. 1A and B, both P<0.001).

### At treatment initiation all animals have disseminated disease

At 36 h post-challenge, bacteria were detected in 100% of the spleens, livers, and lungs irrespective of whether the animals had been immunized with the vaccine formulations. In addition, mice immunized with Alhydrogel and CpG were colonized, 40% (kidney and urine), 20% (brain), and 80% (blood). For mice immunized with the vaccine with CpG, bacteria were detected in 60% of the kidneys and 100% of the brains. For mice that received the vaccine (no CpG), bacteria were detected in 60% of the kidneys and 100% of the brains. Bacteria were found in 100% and 40% of the kidney, and brain samples were harvested from the untreated controls, respectively. Bacteria were detected in 40% of the urine samples from animals immunized with the adjuvant, with no bacteria detected in the urine in any of the other groups. No bacteria were detected in the blood samples of mice that received the vaccine (with or without CpG) in contrast to the mice that received Alhydrogel and CpG (80% colonized) and the untreated mice (60% colonized).

At 48 h post-challenge, all mice were colonized in the spleen, liver, lungs, and kidneys except for one mouse immunized with the vaccine with CpG, which had no bacteria detected in the spleen and liver. Bacteria were detected in the brain in 100%, 100%, 60%, and 80% of samples harvested from mice immunized with Alhydrogel and CpG, the vaccine, the vaccine with CpG and PBS, respectively. Bacteria were detected in the blood in 60%, 20%, and 100% of samples harvested from mice immunized with Alhydrogel and CpG, vaccine (no CpG), and those unvaccinated, respectively. No bacteria was detected in the blood of animals that received the vaccine with CpG. 20%, 40%, 20%, and 20% of urine samples were colonized from mice immunized with the adjuvant, the vaccine (with CpG), the vaccine (without CpG), and untreated mice, respectively.

**Fig 2:**
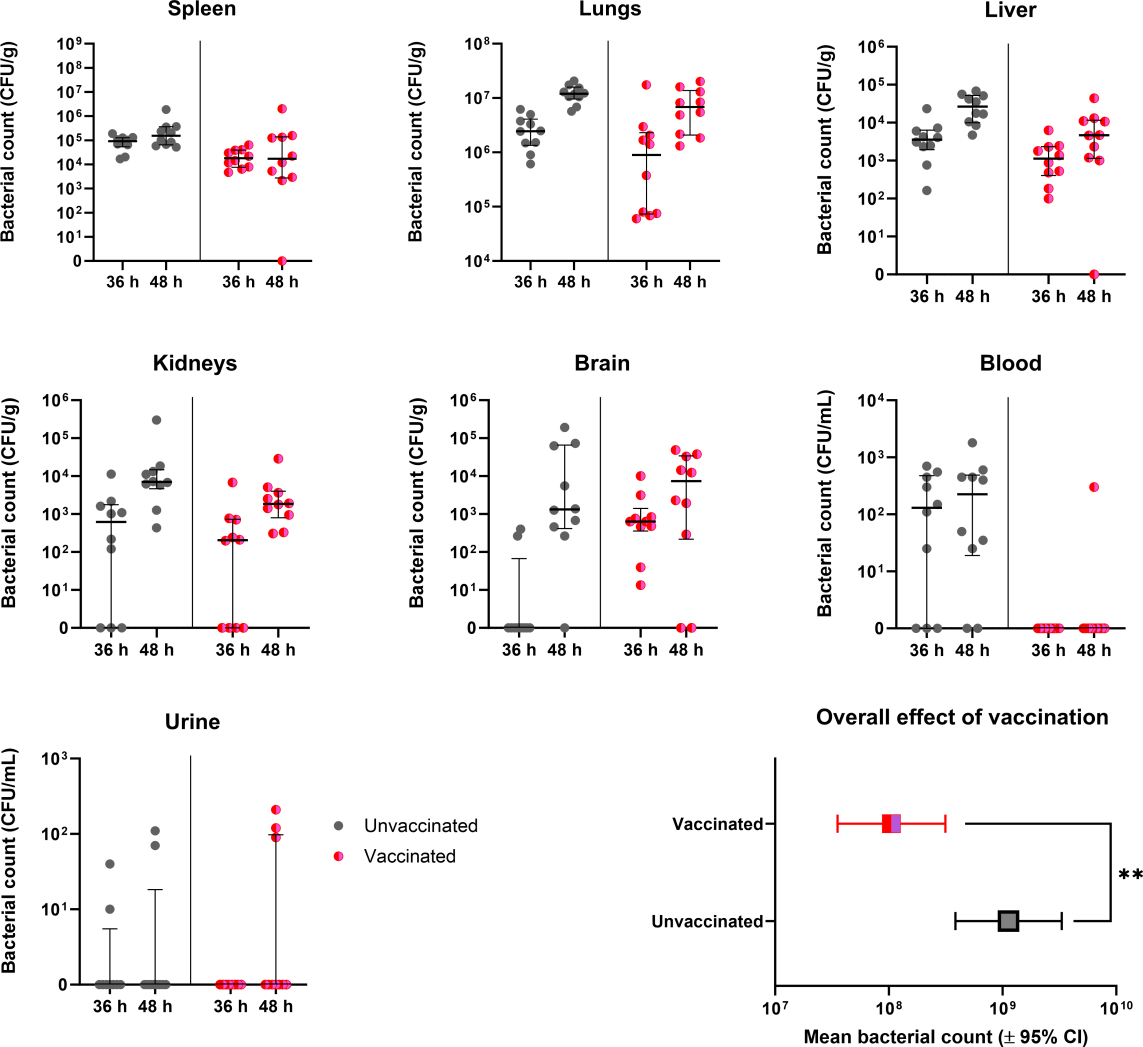
The bacterial load at treatment initiation in vaccinated (with and without CpG) and unvaccinated mice. Mice were vaccinated (as per Table 1) infected with a mean retained dose of 106 CFU of *B. pseudomallei* and groups culled at treatment initiation (36 and 48 h post-challenge) to enumerate the bacterial load in a panel of organs (**P<0.01). Each column contains data from 10 mice, from a single experiment. Urine could not be obtained from three mice.

These data were analyzed using statistical model building. Statistical model building is a process where only descriptive parameters that improve the model’s fit are included. A mixed model was used that accounted for the variance associated with multiple organs originating from the same mouse. The optimal model included the organ, time-point post-challenge (36 or 48 h), vaccine (with and without CpG), and the interaction between time-point and organ. The inclusion of the adjuvant did not benefit the fit of the model (P>0.05). The model found that organ (P<0.001), time (P<0.001), the interaction between organ and time (P=0.127), and vaccine (P=0.003) were required to effectively describe the data. The effect of the vaccine resulted in a reduction in the bacterial load overall (P=0.003, Fig. 2).

### Vaccinated mice demonstrate an extension in time to death compared to the unvaccinated mice

Following vaccination, mice were infected with a mean retained dose of 106 CFU (range 39–157 CFU) of *B. pseudomallei* strain K96243 by the inhalational route, equating to approximately 21 LD_50_ (LD_50_ = approximately 5 CFU). Treatment was initiated at 36 or 48 h post-challenge and continued for 7 days. At 36 and 48 h post-challenge, mice displayed moderate and severe clinical signs of disease, respectively. No animals succumbed to disease before the treatment was initiated. The bacterial challenge was lethal, all control animals (treated with the vehicle) succumbed to infection by day 4 post-challenge.

**Fig 3:**
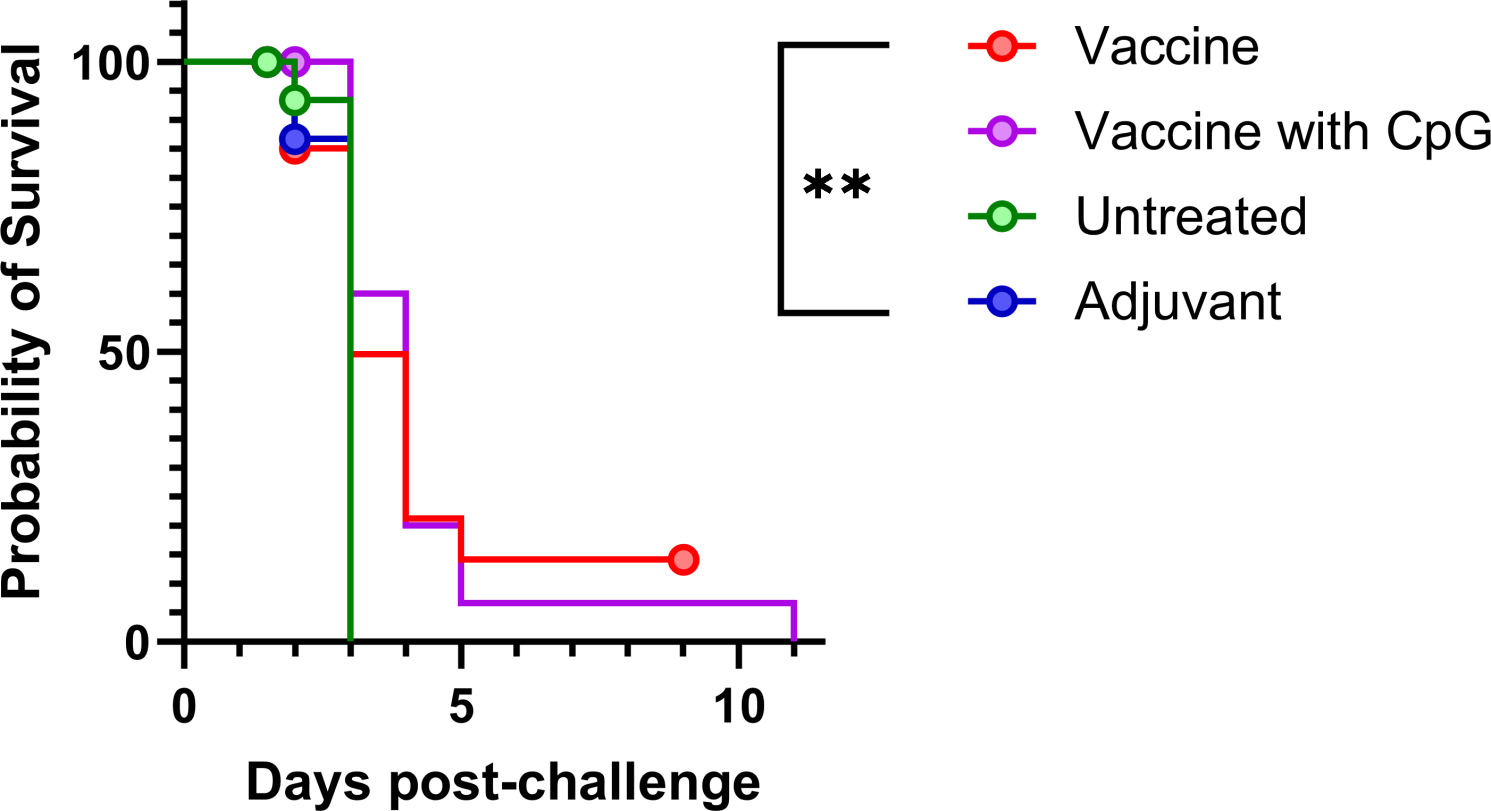
Protection offered by the vaccine formulations following bacterial challenge with *B. pseudomallei*. Each vaccine was compared to the untreated control using log rank tests (adjusted for familywise error) (**P<0.01). The red circle represents two animals that were culled at the experimental timepoint at the end of the treatment period. Other circles represent times when animals were culled for bacterial load analysis (Fig. 2). A single mouse receiving vaccine with CpG survived until day 11 post-challenge (was not part of a scheduled cull group). The group size (including the scheduled cull groups) was *n* = 20 for both unvaccinated groups and *n* = 25 for vaccinated groups. These data are from a single experiment.

Of the animals vaccinated, those that received the vaccine with the CpG component demonstrated a small increase in time to death (P<0.003, when compared to the untreated group) (Fig. 3). The vaccine delivered without CpG did not provide measurable protection (P=0.342 when compared to the untreated group). Additionally, a group was included where mice were treated with the adjuvants but no vaccine. No survival benefit was observed (P>0.999 when compared to the untreated group).

### Vaccination provides additional protection to that offered by delayed finafloxacin monotherapy

**Fig 4:**
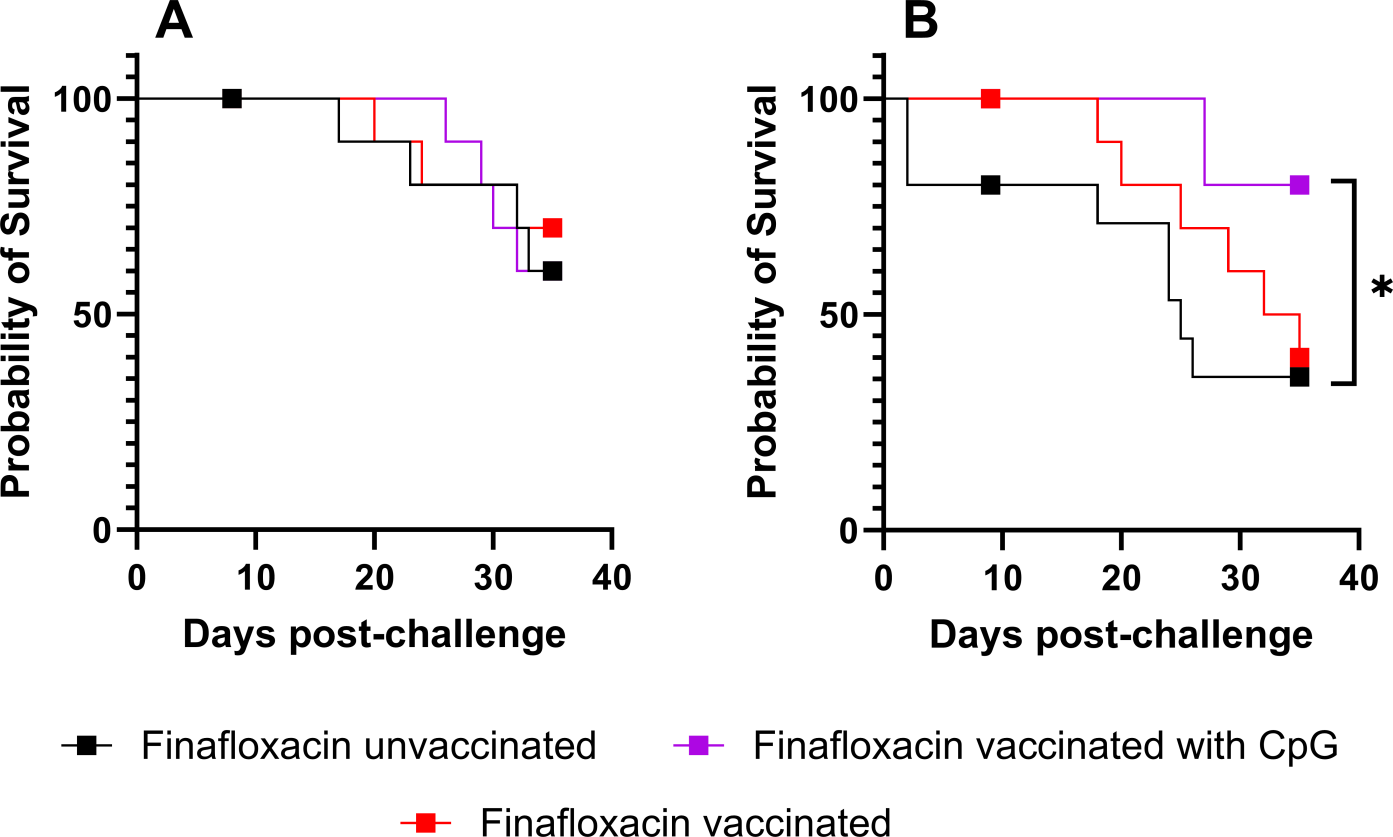
Protection offered by the vaccine and finafloxacin following bacterial challenge with *B. pseudomallei*. Kaplan Meier plots of the survival of mice infected with *B. pseudomallei* and treated with finafloxacin at 36 (**A**) or 48 (**B**) h post-challenge. Some mice were euthanized at predefined time points marked with symbols. The *P*-value is indicative of a logrank test to compare the two groups and adjusted for familywise error (factor of 4). Each vaccination group was compared to the unvaccinated controls (*P<0.05). This study was performed contemporaneously to the work described in Fig. 1 and 2. Each curve is derived from 15 animals and includes groups of 5 mice culled on cessation of the antibiotic to determine the effect on bacterial load (indicated by a square).

Cox regression was used to investigate which parameters influenced survival. Explanatory variables included antibiotic treatment (finafloxacin or diluent), vaccine (vaccinated or PBS vaccinated), and adjuvant (Alhydrogel and CpG or PBS). This analysis indicated a role in survival for antibiotics (P<0.001) and vaccine (P=0.012) but not intervention time (P=0.191) or adjuvant (P=0.674). To determine whether there was any additional protective effect of the vaccine when administered as a component of layered defense, the data were selected and split by treatment time and adjuvant status. This produced four pairs of groups that were compared by logrank tests adjusted for familywise error. The vaccine only provided a measurable synergistic effect when it was delivered with CpG, and finafloxacin was delivered at 48 h post-challenge (P=0.044) (Fig. 4).

### Combining treatment enhances bacterial clearance

All surviving animals were culled at day 35 post-challenge. The bacterial load was determined in spleens, livers, lungs, and blood from survivors. Eighty three percent of spleens, 66% of lungs, and 50% of livers from mice treated with finafloxacin as a monotherapy from 36 h post-challenge were colonized at the end of the study. Adding the full complement of the vaccine (with CpG) to the treatment regimen reduced this to 33% of spleens and livers and 16% of lungs, with the vaccine (no CpG) and finafloxacin treatment resulting in 42% of spleens and lungs and 28% of livers containing detectable bacteria. No bacteria were detected in any of the blood samples, irrespective of group.

All mice treated with finafloxacin as a monotherapy from 48 h post-challenge were colonized, 100% in the spleen, 50% in the lung, and 75% in the liver. Mice immunized with the vaccine (no CpG) and treated with finafloxacin were colonized in the spleen (40%), livers (40%), and lungs (60%). No bacteria were detected in any of the blood samples, irrespective of group. In contrast, all mice immunized with the full complement of the vaccine (with CpG) and treated with finafloxacin were clear of colonizing bacteria.

As detailed above, at the end of the study, no bacteria were found in the blood of surviving animals; however, some mice were still colonized with *B. pseudomallei* within organs. The surviving animals represent a sample biased for animals that tolerated disease. For this reason, a statistical method was used that included the complete data set. All of the animals were assigned a point on an ordinal scale: (i) survived with no detectable bacteria within organs, (ii) survived and were colonized with bacteria, or (iii) culled (due to the mice reaching their humane end point). Model building methodology was used with these scores, using an ordinal, generalized linear model. This is an iterative process whereby a statistical algorithm is used to select explanatory variables that contribute to the model’s fit to the data. The null model included the forced entry of the vaccine as the subject of the hypothesis. Neither the adjuvant or treatment initiation time was found to improve the model fit (P>0.05). Vaccination (with or without CpG) was shown to improve the outcome, i.e., improve clearance and, therefore, survival (P=0.026) (Table 2).

**TABLE 2:** The outcome of mice vaccinated and treated with finafloxacin^*a*^

		Unvaccinated	Vaccinated	Total
Outcome	Survived with no detectable bacteria within organs	n=1 5%↓ 1.7%total	n=18 45%↓ 30%total	n=19 31.7%total
	Survived and colonized with bacteria	n=9 45%↓ 15%total	n=8 20%↓ 13.3%total	n=17 28.3%total
	Culled	n=10 50%↓ 16.7%total	n=14 35%↓ 23.3%total	n=24 40%total
Total		n=20 33.3%total	n=40 66.7%total	n=60 100%total

^*a*^ The outcome data from Fig. 4 (not including those mice culled to after cessation of antibiotics) were analyzed by an ordinal general linear model. Data are shown as the frequency (*n*), the percent column total (↓), and the percent overall total (*total*). This includes vaccine data with and without CpG and finafloxacin initiated at 36 and 48 h post-challenge. Neither the treatment time or the addition of CpG impacted on this model and is not included in these frequencies.

### MICs on bacteria harvested from surviving animals

MICs were performed on bacteria isolated from surviving animals treated with finafloxacin. No evidence of resistance was observed as the MICs were comparable to the original strain of *B. pseudomallei* used in the study (4 µg/mL).

## DISCUSSION

Melioidosis is a difficult disease to treat, requiring lengthy courses of antibiotics, and even if the treatment regimen is successfully completed, there is still a risk of relapse of infection later on in life due to the antibiotics not completely clearing the bacterium from the host (41). In addition, the identification of *B. pseudomallei* from the environment in new geographical locations including the USA and the emergence of strains that are resistant to the current therapeutic options (including ceftazidime and co-trimoxazole) makes it a significant public health and defense concern (3, 42, 43).

The investigation of different approaches to provide improvements or alternatives to current treatment options is warranted, including the use of combination therapy or layered defense strategies. Previously, we have demonstrated the benefit of combining finafloxacin and doxycycline to treat *B. pseudomallei* infections in mice (33). Building on this concept, it was hypothesized that vaccination with a CPS-based subunit vaccine may aid with bacterial clearance (following a *B. pseudomallei* inhalational exposure but prior to antibiotic treatment being initiated). Conceptually, the vaccine-primed immune system will respond to and kill the bacterial cells by a mechanism that is different from the antibiotic activity. The aim of this study was to investigate the effect of layering this vaccine with the antibiotic finafloxacin on the level of protection offered, the clearance of colonizing bacteria from tissues, and the relapse of infection in Balb/c mice when the treatment initiation time was delayed to 36 and 48 h post-challenge.

As described above, groups of mice vaccinated and infected were euthanized at antibiotic initiation and the bacterial load enumerated in a panel of tissues. Overall, the vaccinated animals had lower levels of bacteria in the tissues compared to those that had not been immunized. Interestingly, although the group sizes were small (*n* = 5), no bacteria was detected in the blood at 36 or 48 h post-challenge in mice immunized with the vaccine with CpG. This indicates that the resulting immune response was able to reduce the amount of bacteria circulating in the blood. The high level of IgG generated to both vaccines (immunized with and without CpG) and both antigens (CPS and Hcp1) provide evidence for the presence of components of the adaptive immune response in vaccinated animals and may help clear *B. pseudomallei* from the lower respiratory tract, known to contain high levels of IgG (44). The protection afforded by this vaccine, in the absence of antibiotic, was minimal but measureable. This is likely due to the often reported immune bias of Balb/c mice toward Th2, whereas protection against melioidosis is known to benefit from a more cell-mediated, Th1-biased response (45, 46).

Finafloxacin delivered as a monotherapy provided 60% and 40% protection when administered at 36 and 48 h post-challenge, respectively. This level of protection offered by the monotherapy is encouraging considering that this was a short course (7 days) of treatment, which is suboptimal (14 days of treatment initiated at 36 h post-challenge in a previous study offered 90% protection) (33). In addition, the treatment was initiated when animals were showing clinical signs of disease, the bacterial load was 10^4^–10^7^ CFU/g in the lungs, and there were bacteria in the blood, indicative of disseminated disease. Layering of the CPS-based subunit vaccine to this antibiotic did appear to make a difference, providing a significant difference in the level of protection (80%) offered when compared to finafloxacin treatment (delivered at 48 h post-challenge).

Similar levels of protection and bacterial clearance in tissues have been previously demonstrated with the CPS-based subunit vaccine and the antibiotic co-trimoxazole, in the C57BL/6 mouse, an animal model of melioidosis which is considered more resistant to *B. pseudomallei* infections and is typically used for the evaluation of vaccines (35, 47). The data detailed in this manuscript are particularly encouraging, considering that the time post-challenge the antibiotic treatment was initiated, was very late and a suboptimal finafloxacin regimen was utilized. In addition, the Balb/c mouse is a very sensitive model, requiring only 5 CFU by the aerosol route to establish an infection which rapidly disseminates (15).

One interesting observation is that all organs from surviving animals immunized with the vaccine with CpG and treated with a suboptimal dose of finafloxacin from 48 h post-challenge were clear of colonizing bacteria, whereas those mice treated from 36 h post-challenge were not all clear. This may be due to components of the immune system requiring activation to respond to the bacterial challenge, and by 48 h, both the innate and adaptive immune response are completely functional. When bacteria invade an intracellular niche, it is the antibodies that are predominantly protective before the bacteria start to replicate; therefore, the combination of the antibody response with the antibiotic treatment could have killed a proportion of the *B. pseudomallei* cells before it was able to invade and replicate within cells (48). It has been reported that during the early stage of the immune response, only a proportion of the effector T cells that enter tissues colonized with bacteria will be specific for the infecting pathogen and that successful clearance of an infecting organism is dependent on interactions between antigen-specific T-cells and infected macrophages (49, 50) Although high titers of IgG were generated against both Hcp1 and CPS, it is well documented that both T and B cells are required to respond against *B. pseudomallei* infections. Future studies would include an investigation into the T cell responses in vaccinated mice.

The data detailed in this manuscript suggest that CpG is a very important component of the CPS-based subunit vaccine. Although no protection was demonstrated with the vaccine (without CpG) in the Balb/c mouse model, the addition of the CpG component provided a small increase in time to death and was able to further stimulate the immune system to produce a significantly increased IgG titer, one of its well-documented functions being an agonist of Toll-like receptor 9 (51, 52). Another CpG, 1018, has been approved by the FDA as a component of a hepatitis vaccine; therefore, there is precedent for including a CpG adjuvant as a component of a vaccine for use in humans (53).

In summary, the inclusion of the CPS-based subunit vaccine with a suboptimal finafloxacin treatment regimen had a positive effect on reducing the bacterial load in tissues before treatment initiation and provided a synergistic effect when it was delivered with CpG and finafloxacin was initiated at 48 h post-challenge. Vaccination was also shown to improve the outcome, i.e., it enhanced bacterial clearance and, therefore, survival in a mouse model of melioidosis. This has demonstrated that layering a CPS-based subunit vaccine with the antibiotic finafloxacin is a promising alternative therapeutic strategy to be used in the treatment of *B. pseudomallei* infections. In the future, it would be interesting to delay the time the antibiotic was initiated further and also reduce the dose or number of doses of vaccine-administered pre-exposure to investigate the effect of dose sparing on the parameters described above.
